# Oligonucleotide Binding to Non-B-DNA in *MYC*

**DOI:** 10.3390/molecules24051000

**Published:** 2019-03-12

**Authors:** Tea Umek, Karin Sollander, Helen Bergquist, Jesper Wengel, Karin E. Lundin, C.I. Edvard Smith, Rula Zain

**Affiliations:** 1 Department of Laboratory Medicine, Clinical Research Center, Karolinska Institutet, Karolinska University Hospital Huddinge, 141 86 Huddinge, Sweden; hebe7619@gmail.com (H.B.); karin.lundin@ki.se (K.E.L.); edvard.smith@ki.se (C.I.E.S.); 2 Department of Molecular Biology and Functional Genomics, Stockholm University, 171 65 Stockholm, Sweden; karin.sollander@scilifelab.se; 3 Biomolecular Nanoscale Engineerng Center, Department of Physics, Chemistry and Pharmacy, University of Southern Denmark, M5230 Odense, Denmark; jwe@sdu.dk; 4 Department of Clinical Genetics, Centre for Rare Diseases, Karolinska University Hospital, SE-171 76 Stockholm, Sweden

**Keywords:** MYC, non-B-DNA, H-DNA, G-quadruplex, anti-gene oligonucleotide

## Abstract

*MYC*, originally named c-*myc*, is an oncogene deregulated in many different forms of cancer. Translocation of the *MYC* gene to an immunoglobulin gene leads to an overexpression and the development of Burkitt’s lymphoma (BL). Sporadic BL constitutes one subgroup where one of the translocation sites is located at the 5’-vicinity of the two major *MYC* promoters P_1_ and P_2_. A non-B-DNA forming sequence within this region has been reported with the ability to form an intramolecular triplex (H-DNA) or a G-quadruplex. We have examined triplex formation at this site first by using a 17 bp triplex-forming oligonucleotide (TFO) and a double strand DNA (dsDNA) target corresponding to the *MYC* sequence. An antiparallel purine-motif triplex was detected using electrophoretic mobility shift assay. Furthermore, we probed for H-DNA formation using the BQQ-OP based triplex-specific cleavage assay, which indicated the formation of the structure in the supercoiled plasmid containing the corresponding region of the *MYC* promoter. Targeting non-B-DNA structures has therapeutic potential; therefore, we investigated their influence on strand-invasion of anti-gene oligonucleotides (ON)s. We show that in vitro, non-B-DNA formation at the vicinity of the ON target site facilitates dsDNA strand-invasion of the anti-gene ONs.

## 1. Introduction

DNA is a dynamic macromolecule, which can adopt various conformations. The right-hand double helix B-DNA, is believed to be the most frequent structure in vivo [1]. However, evidence for the possible presence of several alternative DNA conformations has emerged, such as Z-DNA, intramolecular triplex (H-DNA), cruciform and G-quadruplex. These DNA structures are collectively called non-B-DNA and their implication in different biological processes, has been reported, in particular in relation to disease-associated genes. For example, non-B-DNA forming sequences induce genetic instability leading to mutagenic hotspots in the genome [2] such as in the *MYC* gene. Defective regulation of *MYC* leads to several forms of cancers. High levels of the MYC protein allow tumour initiation, progression and maintenance [3], which is associated with Burkitt’s lymphoma (BL) [4], among other cancer diseases. BL has been divided into three different subgroups: endemic, sporadic and immunodeficiency-associated. Patients in these categories differ in their geographic location and age of disease onset among other things (Supplementary Table S1) [5,6,7].

**Figure 1:**
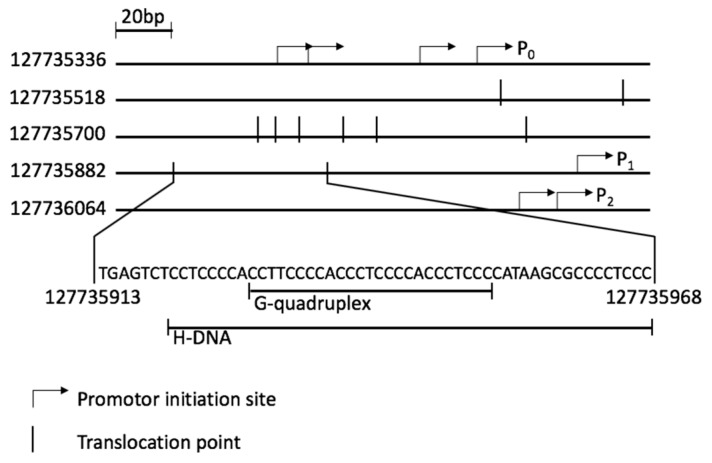
Promoter region of the *MYC* gene (127735336 to 127736236 *Homo sapiens* chromosome 8, according to assembly GRCh38.p12) showing initiation sites of the three promoters P_0_–P_2_, NHEIII_1_ sequence and a cluster of translocation points (vertical lines). P_0_–four initiation sites are indicated. P_1_–one initiation site and P_2_–two initiation sites are also shown.

The MYC protein, which forms heterodimers with either MAX or MLX, is involved in many essential pathways within the cell, such as proliferation, growth and apoptosis [8]. The cellular level of MYC is crucial for the correct regulation of these pathways. Its expression is regulated during transcription and translation. Additionally, it depends on the stability of the mRNA and protein [9]. The *MYC* gene, with two open reading frames, is transcribed from four promoters: P_0_, P_1_, P_2_ and P_3_, of which P_1_ and P_2_ are the two major ones (Figure 1) [10,11]. A nuclease hypersensitivity element III_1_ (NHEIII_1_) [10], located upstream of P_1_, correlates with transcriptional activity of both P_1_ and P_2_ [12]. It has been shown that a non-B-DNA structure, either H-DNA or G-quadruplex, could be formed within this sequence [13]. Both conformations are associated with blocking DNA replication and transcription [14,15,16]. Additionally, they increase the frequency of breaks in double strand (dsDNA), thereby inducing translocation events [17]. Furthermore, enhanced stability of H-DNA results in increased mutational frequency [2]. In BL the translocation event involves the *MYC* gene and one of the three immunoglobulin (IG) genes, *IGH*, *IGK* or *IGL* [17,18]. Mapping of translocation events in BL patients presents a cluster of translocation points surrounding the NHEIII_1_ (Figure 1), more specifically 5’ of the P_1_ promoter, in the first exon and in the first intron (Supplementary Figure S1) [19].

**Figure 2:**
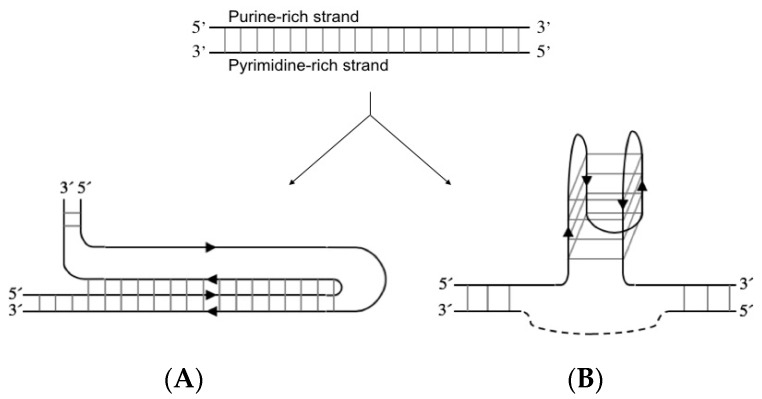
Schematic representation of a purine-rich/pyrimidine-rich sequence forming (**A**) purine- motif intramolecular triple-helix structure (H-DNA) or (**B**) antiparallel intramolecular G-quadruplex structure where the pyrimidine-rich strand (dotted line) can also adopt an i-motif (not shown). Formation of G-quadruplex and i-motif is considered mutually exclusive [25,26].

H-DNA is an intramolecular triplex structure that can form at polypurine/polypyrimidine sequences through the formation of Hoogsteen or reverse Hoogsteen hydrogen bonds between the bases (Figure 2A) [20,21]. Negative supercoiling of plasmids affects the ability of these sequences to generate an intramolecular triplex. In vivo, the structure is proposed to be formed due to negative supercoiling following replication and transcription [22]. The G-quadruplex is formed by a polypurine strand containing at least four guanosine (G)-stretches. This strand can either fold into four parallel or antiparallel G-rich stretches, oriented in a quadrate pattern stabilised by hydrogen bonds between the bases (Figure 2B). The intramolecular structure is additionally stabilised by monocations localised between the sheets of four interacting G bases [23,24]. The G-quadruplex can also consist of two or four separate G-rich strands.

Both H-DNA and G-quadruplex can form in sequences within several oncogenes and, thereby, are associated with various cancer diseases, making them a potential target for therapy [27,28,29]. In contrast to current chemotherapeutic drugs, that affect dsDNA nonspecifically [30], selective targeting of non-B-DNA structures could reduce off-target effects. To this end, low-molecular weight compounds have been designed and examined [31,32,33]. Moreover, a potential novel strategy relates to the use of anti-gene oligonucleotides (ONs), which bind dsDNA in a sequence-specific manner. The majority of such ONs are triplex forming ONs (TFOs) [34,35,36] and peptide nucleic acids (PNAs) [35,37,38]. They modulate *MYC* expression to prevent cancers caused by overexpression of the gene. Although several TFOs and PNAs have been designed against the *MYC* gene, very few are directed towards the non-B-DNA sequence [39].

Anti-gene ONs have also been developed to bind DNA through an invasion mechanism where PNA oligomers and ONs based on locked nucleic acid (LNA) have been used [40,41,42,43]. Strand-invasion of dsDNA occurs when using LNA ONs or PNAs having enhanced binding affinity for complementary strands compared to unmodified ONs [44,45,46]. This results in the formation of new Watson-Crick hydrogen bonds between the ON and one strand of the DNA duplex. Consequently, the opposite DNA strand is then displaced. Reports have shown that PNA can both prevent and induce the formation of G-quadruplexes [47]. Bergquist et al., have demonstrated that PNA and LNA ONs can, by strand-invasion, disrupt H-DNA structures within expanded triplet-repeats, present in various diseases, such as in Friedreich’s ataxia [48]. To increase the stability of PNA- or LNA-based DNA complexes, clamp type strand-invading ONs have been developed, so called bisPNA and bisLNA, respectively. Such ON constructs utilize two modes of binding, simultaneously forming a duplex and a triplex with one strand of the dsDNA target. In bisLNA, two ONs are connected by a linker where one binds as a TFO forming Hoogsteen hydrogen bonds and the second via WC base pairing [41]. Under physiological pH and salt conditions, LNA-based clamp type ONs (bisLNAs) can recognize polypurine/polypyrimidine sequences with high specificity and form stable triplexes able to withstand DNA relaxation [40,41].

Here we have examined triplex formation using a non-modified DNA TFO to identify the most favoured triplex motif that can be formed in this sequence. Also, we probed H-DNA formation, within the NHEIII_1_ in *MYC*, using the triplex-specific cleavage assay of dsDNA, based on a benzoquinoquinoxaline 1,10-phenanthroline compound (BQQ-OP) [49,50]. This cleaving agent constitutes a triplex-specific intercalating benzoquiniquinoxaline compound (BQQ) conjugated to a nucleic acid cleaving moiety (5-methylphenanthroline). Moreover, we have monitored DNA strand-invasion (DSI) using bisLNA, which targets a sequence at the vicinity of the H-DNA forming site. We have assessed the effect of H-DNA formation on the efficiency of the bisLNAs to carry out strand-invasion under intranuclear salt-conditions in vitro and to bind this particular site in the *MYC* promoter region.

## 2. Results and Discussion

### 2.1. Intermolecular Triplex Is Formed with Pu TFO But Not with Py TFO

Electrophoretic mobility shift assay (EMSA) was performed with either purine-rich DNA TFO (Pu TFO) or a pyrimidine-rich DNA TFO (Py TFO) hybridized with the target sequence to evaluate intermolecular triplex structure formation. Both Pu- and Py-TFOs have a central interruption of a T and an A, respectively, to mimic the situation where an H-DNA structure would form in the genomic sequence. The gel mobility of the dsDNA target and the ON:dsDNA complex depends on their size, charge and structure, where a triplex DNA has a slower mobility than a duplex. The formation of intermolecular triplex was analysed using different ratios of target to TFO. Hybridization was performed using different pH (6.5 for Py TFO and 7.4 for Pu TFO) and cations (Na^+^ or Mg^2+^) concentrations to establish favourable conditions for pyrimidine- and purine-motif triplex formation, respectively.

**Figure 3:**
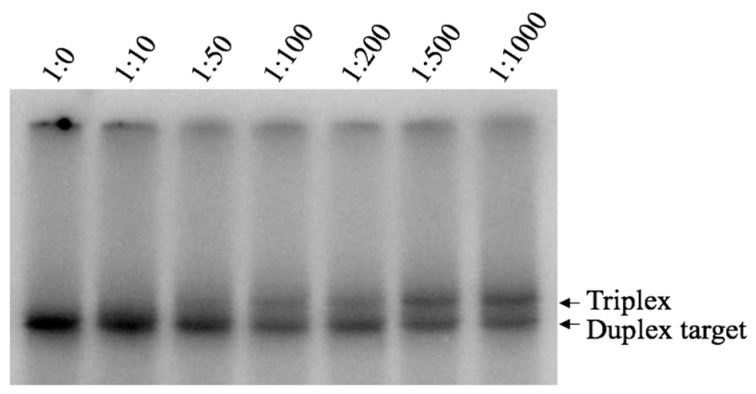
EMSA analysis of DNA Pu-TFO binding to target dsDNA. Ratios dsDNA:Pu-TFO 1:0–1:1000 are indicated on top and lane 1:0, where only the duplex DNA is present is used here as a reference. DNA binding was carried out during 19 h at 4 °C and pH 7.4 in presence of 10 mM Mg^2+^. The samples where anaylsed using non-denaturing polyacrylamide gel electrophoresis. Arrows indicate bands representing the duplex and triplex, respectively.

Binding of Pu TFO to the dsDNA target was detected as shifted band corresponding to triplex formation, which was first visible at ratio 1:100; however, the intensity of this band increased gradually when higher TFO concentrations were used (Figure 3, lane 1:500, 1:1000). These results strongly indicate the formation of an intermolecular triplex with the purine-rich third strand under near physiological pH and salt conditions. Interestingly, the purine-motif triplex is formed despite the presence of a one-base interruption in the polypurine/polypyrimidine sequence. The Py TFO did not form any detectable structure (data not shown). Our results indicates a favoured purine-, but not pyrimidine-, motif triplex, which is in agreement with previous publications [39].

### 2.2. BQQ-OP Cleavage of pMycNHE+ Detects the Presence of H-DNA

**Figure 4:**
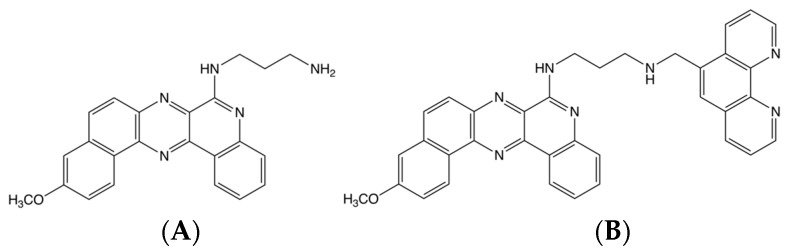
Chemical structure of (**A**) BQQ and (**B**) BQQ-OP.

Benzoquinoquinoxaline (BQQ) is a heterocyclic compound with an aminoalkyl side chain, which has previously been shown to preferentially bind to DNA triplex structures [51,52]. BQQ intercalates between the bases, thus, stabilising the triplex conformation. Conjugation of BQQ to 1,10-phenanthroline (*ortho*-phenanthroline, OP) was previously carried out leading to the triplex-specific cleaving agent, abbreviated BQQ-OP, which specifically binds and cleaves double strand DNA at the site of formation of a triplex structure [50]. The chemical structure of both molecules can be seen in Figure 4. BQQ-OP cleaves the DNA in the presence of Cu^2+^-ions, which chelates to OP, and a reducing agent producing in situ radicals. BQQ-OP enables structural analyses of triplex structures and is here used to detect H-DNA within the NHEIII_1_ sequence in the plasmid, pMycNHE+.

**Figure 5:**
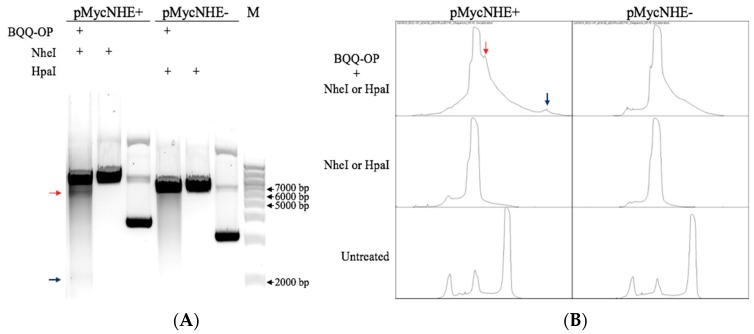
BQQ-OP cleavage indicating H-DNA formation in pMycNHE+. (**A**) BQQ-OP mediated cleavage of pMycNHE+ and pMycNHE- was carried out in the presence of Cu^2+^ and MPA. Both plasmids were further treated with a unique-site restriction enzyme. Red and blue arrows mark the bands, 5447 and 2214 bp long, indicating H-DNA site cleavage. Reference linearized plasmids, untreated plasmids and a molecular weight DNA ladder (M) are also shown. (**B**) Lane profile plots of the BQQ-OP plus restriction enzyme-cleaved plasmid, linearized plasmid (NheI or HpaI), and supercoiled (untreated) plasmid. Blue and red arrows indicate peaks corresponding to the equally marked bands in (**a**).

Both, pMycNHE+ and the corresponding control plasmid, pMycNHE- (Supplementary Table S2), were incubated with BQQ-OP in the presence of Cu^2+^ and mercaptopropionic acid (MPA). BQQ-OP cleavage was followed by DNA digestion using a single-site specific restriction enzyme, which in the case of triplex-specific BQQ-OP cleavage should result in two DNA fragments corresponding to average sizes of 5447 bp and 2214 bp. As seen in Figure 5A, the pMycNHE+ lanes show, additionally to the band corresponding to linear plasmid, two faint bands, marked with colored arrows, indicating BQQ-OP mediated cleavage of the plasmid. This is further confirmed with the lane profile plots (Figure 5B), generated with ImageJ. The blue and red arrows in Figure 5B, indicate the peaks that correspond to the arrow-marked bands in Figure 5A. As seen from the plots, no such peaks are observed in the pMycNHE- control plasmid, or in the restriction enzyme-only treated or untreated plasmids. Taken together, an H-DNA structure is formed at this specific *MYC* promoter sequence.

### 2.3. Influence of H-DNA Formation on bisLNA Mediated dsDNA Strand-Invasion

The S1 nuclease assay was used to determine the strand-invading efficiency of bisLNAs and of a Watson-Crick (WC) binding ON in the presence or absence of H-DNA, thereby analysing the effect of the structure on the strand-invasion event. The S1 enzyme is an endonuclease that recognises single-stranded DNA or RNA and degrades it, leaving 5’-phosphoryl-terminated products. Double-stranded nucleic acids are resistant to S1 activity. In theory, hybridization of bisLNA to the complementary sequence in the plasmid and subsequent strand-invasion, leaves the opposite strand exposed to S1 nuclease activity. The enzyme creates a nicked or linearized plasmid, which can be observed by the different migration patterns in the agarose gel compared to the supercoiled plasmid.

**Figure 6:**
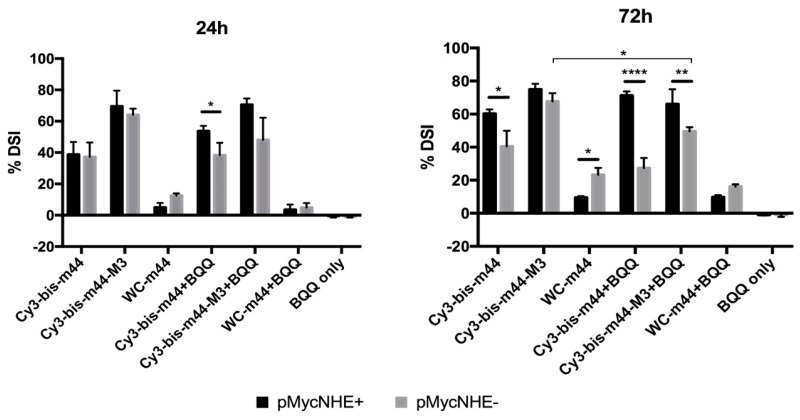
Strand-invasion of two bisLNAs and the corresponding WC-ON after 24 or 72 h, as detected by the S1 nuclease assay. The concentration of the ONs is 4.05 µM. The percentage is calculated from the ratio of nicked plasmid to the total amount of plasmid and normalized to the same ratio in mock treated plasmid. Statistical analysis done with Sidak’s multiple comparisons test (**** *p* < 0.0001, *** *p* < 0.001, ** *p* < 0.01, * *p* < 0.05).

To this end, three ONs were chosen. BisLNA with a 5 nt (nucleotide) linker (Cy3-bis-m44), bisLNA with a DNA intercalating linker (Cy3-bis-m44-M3) and a WC arm of the bisLNA (WC-m44). The ONs were incubated with the plasmids for 24 h or 72 h (Figure 6). Furthermore, ONs were also hybridized in the presence of BQQ, the DNA triplex-stabilising compound. The percentage of DSI at 24 h per ON is similar for both plasmids except for Cy3-bis-m44 in the presence of BQQ. As previously shown [41], bisLNA with an intercalating M3 linker has higher strand-invasion capability than the ON with 5 nt linker at 24 h. Cy3-bis-m44-M3 reaches the plateau already after 24 h, as the percentage of DSI is similar after 72 h. Contrary, the Cy3-bis-m44 has a slower mode of action as the DSI increases over time; however, only for pMycNHE+, which indicates that H-DNA has some influence on the invasion. This could be further supported by hybridizations carried out in the presence of BQQ. The statistical difference can already be observed after 24 h for Cy3-bis-m44+BQQ. After 72 h, the difference between the plasmids increases, especially in the presence of BQQ since the percentage of DSI in the pMycNHE+increase or remains high, while in the pMycNHE- the DSI decreases, suggesting that H-DNA instigates the invasion. It must be considered that the H-DNA structure, formed at the *MYC* sequence is highly unstable in the absence of stabilizing conditions. Therefore, the mode of action observed in the presence of BQQ does not readily translate to in vivo conditions; however, it might give some indication, if the structure would be stabilized by DNA binding metabolites or proteins. In contrast to both bisLNAs, the WC LNA-ON shows better strand-invasion in the absence of H-DNA, although this could be due to the particular sequence of this ON being able to additionally bind as a TFO, which would not be detected by this assay.

Previous studies indicated a possible connection between the formation of a non-B-DNA structure within the *MYC* promoter region and translocation of the gene in Burkitt’s lymphoma [2,17,53]. We have been able to determine that an intermolecular purine-motif triplex structure can be formed in the selected 17 bp sequence of the *MYC* promotor region, whereas no structure could be detected when using a pyrimidine-rich TFO. We further identified an intramolecular structure, specifically an H-DNA, in the plasmid containing a larger fragment of the *MYC* promoter region. Finally, we have evaluated the influence of the H-DNA on the strand-invading capability of anti-gene LNA-ONs, which can potentially be used in a therapeutic setting [54]. It is suggested that H-DNA contributes to ON-mediated strand-invasion and reduces the dissociation of the corresponding ONs.

## 3. Materials and Methods

### 3.1. TFO Design and Electrophoretic Mobility Shift Assay (EMSA)

We designed a TFO with a sequence able to form an intermolecular triple-helix structure that covers the site of the tandem H-DNA [55] and the G-quadruplex models [56,57]. The dsDNA target, Pu TFO and Py TFO (17 bp) (Table 1) were obtained from Thermo Fischer Scientific, Waltham, MA USA. The pyrimidine-rich strand of the target sequence was ^32^P labelled at the 5’-end using T4-kinase according to the manufacturer’s instruction (Fermentas, Waltham, MA USA). The purine target strand sequence is interrupted with a pyrimidine base, T. Since the pyrimidine base is unable to form Hoogsteen bonds with the third strand, a mismatch will occur between the target and the TFO.

EMSA was used to analyze the formation of intermolecular triplex structures between the duplex target sequence and TFOs. Five nM labelled target was incubated with different concentrations of unlabelled TFO, ranging from 0**–**5 µM. The mixture was incubated for 19 h, at 4 °C. Na^+^ and Mg^2+^ were added at concentrations: 0 or 100 mM and 0 or 5 mM respectively. The pH was adjusted to 6.5, for samples binding a Py TFO and 7.4, for Pu TFO. The samples were analysed on a 10% or 15% non-denatured polyacrylamide gel, run at 150 V for 3**–**5 h. The ^32^P signal was detected using a Fuji FLA3000 phosphorimager and analysed with Multi Gauge v3.0 Software (Fujifilm, Minato, Japan).

### 3.2. BQQ-OP-Cleavage

Triplex formation was verified using the BQQ-OP cleavage assay [50]. Plasmids (Supplementary Table S2) pMycNHE+ (bisLNA target site positioned 11 bases upstream of the NHEIII_1._ The NHEIII_1_ is located in the *MYC* gene at position 127735920–127735968 according to assembly GRCh38.p12) and pMycNHE- containing a bisLNA target site but lacking the NHE sequence, (in [40] named pEGFPLuc-bisBSf) were incubated in 10 mM cacodylate buffer containing 100 mM Na^+^, pH 6.5 together with BQQ-OP and Cu^2+^ for 45 min at RT. Triplex-specific dsDNA cleavage was induced with 2 mM mercaptopropionic acid at 37 °C for 3 h. After, the DNA was isolated with PCR purification kit (QIAGEN, Hilden, Germany) and total DNA was cleaved with *Nhe*I or *Hpa*I (Fast digest). The samples were then loaded on freshly prepared 0.7% agarose gels in 0.5x Tris-Boric-Acid-EDTA buffer (TBE) (0.05 M Tris, 0.045 M Boric Acid, 0.5 mM EDTA) (Invitrogen, Carlsbad, CA, USA), containing 1x SYBRGold (Invitrogen, Carlsbad, CA, USA). The gels were run at 70 V for 3 h and analysed with a VersaDoc Imaging System (Bio-Rad, Hercules, CA, USA) using the QuantityOne software (Bio-Rad, Hercules, CA, USA).

### 3.3. bisLNA Strand-Invasion

pMycNHE+ or pMycNHE- (1 μg, 100 ng/μL) (Supplementary Table S2) were hybridised with bisLNAs Cy3-bis-m44, Cy3-bis-m44-M3 or WC arm (WC-m44) [41] at final concentration of 4.05 μM (the ON-sequences can be found in Table 2). BQQ was added 60 min prior to the addition of ONs to stabilize the H-DNA. The final concentration of buffer added corresponds to intra-nuclear salt conditions (50 mM tris-acetate (pH 7.3–7.4), 120 mM KCl, 5 mM NaCl and 1 mM Mg(OAc)_2_). The hybridizations were carried out in 10 μL total volume in an oven incubator for 24 or 72 h at 37 °C. The S1 Nuclease Assay [58] was used to determine the ONs’ strand-invasion efficiency. Plasmid (250 ng), pre-hybridized with ON was digested with 24 U of S1 nuclease (Promega, Madison, WI, USA) in 1× S1 buffer (Promega, Madison, WI, USA) and water in a final volume of 11 μL. The reaction was performed on wet ice and terminated after exactly 6 min with 3 μL of 0.5 M EDTA. After 10 min, 1.5 μL of the reaction was mixed with 3.5 μL 1× TE buffer (QIAGEN, Hilden, Germany) and 1 μL 6× Orange DNA Loading Dye (Thermo Fisher Scientific, Waltham, MA USA), and the 6 μL total volume was loaded on a freshly prepared 0.9% agarose gel in 0.5× Tris-Boric-Acid-EDTA buffer (TBE) (0.05 M Tris, 0.045 M boric acid, 0.5 mM EDTA) (Invitrogen, Carlsbad, CA, USA), containing 1× SYBRGold (Invitrogen, Carlsbad, CA, USA). The gel was run at 90 V for 60 min and analysed with a VersaDoc Imaging System (Bio-Rad, Hercules, CA, USA) using the QuantityOne software v4.6.9 (Bio-Rad, Hercules, CA, USA). Percentage of double-strand invasion was determined after agarose gel electrophoresis by calculating the ratio of nicked plasmid to the total plasmid amount in a sample and normalized to the same ratio in the mock hybridized sample.

## Figures and Tables

**Table 1:** Sequences of double-strand DNA target and TFO ONs used in EMSA experiments ^1^. The TFOs target parts of both the H-DNA and G-quadruplex forming sequences.

**Target**	5′GTCTCCTCCCCACCTTCGCGACCCTCCCCACCCTCCCCATAAGCGCCCCTCCCGGGTTCC3′ 3′CAGAGGAGGGGTGGAAGCGCTGGGAGGGGTGGGAGGGGTATTCGCGGGGAGGGCCCAAGG5′
Pu TFO	5′GGGAGGGGTGGGAGGGG 3′
Py TFO	3′CCCTCCCCACCCTCCCC 5′

^1^ Bases marked in red in the dsDNA target sequence indicate base shift compared to the genomic sequence, made to avoid unspecific binding. The purine target strand sequence is interrupted with a pyrimidine, marked in bold. TFO and target sequences are aligned to visualize the binding site.

**Table 2:** List of ONs and corresponding sequences ^2^.

ON	Sequence 5′–3′
Cy3-bis-m44	Cy3-CcTtTtCtTtTtTcT-*tctct*-tCtTtTtTcTtTtCcCccAcgCccTctGc
Cy3-bis-m44-M3	Cy3-CcTtTtCtTtTtTcT-*M3*-tCtTtTtTcTtTtCcCccAcgCccTctGc
WC-m44	tCtTtTtTcTtTtCcCccAcgCccTctGc

^2^ LNA bases are in capital letters; DNA bases are in small letters. The linker is in italic.

## Supplementary Materials

The following are available online at https://www.mdpi.com/1420-3049/24/5/1000/s1, Figure S1: 41965761-41966661 Homo sapiens chromosome 8, Table S1: Summary of BL subgroups, Table S2: plasmids pMycNHE+ and pMycNHE- and non-B- DNA and target sequence, respectively. References [5,6,7,17,40,59] are cited in the supplementary materials.
