# Supplementary material for: Oligonucleotide Binding to Non-B-DNA in MYC
Source: Molecules. 2019 Mar 12;24(5):1000. doi: 10.3390/molecules24051000 (PMC6429085; doi:10.3390/molecules24051000)
Supplement: Supplementary file 1 [file molecules-24-01000-s001.pdf]

Supplementary Materials

Oligonucleotide Binding to Non-B-DNA in *MYC*

Tea Umek [1,*], Karin Sollander [2], Helen Bergquist [1], Jesper Wengel [3], Karin E. Lundin [1], C.I. Edvard Smith [1] and Rula Zain [1,4,*]

[1] Department of Laboratory Medicine, Clinical Research Center, Karolinska Institutet, Karolinska University Hospital Huddinge, 141 86 Huddinge, Sweden; karin.lundin@ki.se (K.E.L.); edvard.smith@ki.se (C.I.E.S.); hebe7619@gmail.com (H.B.)

[2] Department of Molecular Biology and Functional Genomics, Stockholm University,171 65 Stockholm, Sweden; karin.sollander@scilifelab.se

[3] Biomolecular Nanoscale Engineerng Center, Department of Physics, Chemistry and Pharmacy, University of Southern Denmark; Odense M, 5230, Denmark; jwe@sdu.dk

[4] Department of Clinical Genetics, Centre for Rare Diseases, Karolinska University Hospital, SE-171 76 Stockholm, Sweden

* Correspondence: tea.umek@ki.se (T.U.), rula.zain@ki.se (R.Z.); Tel.: +46-(0)8-5858 3663 (T.U.), Tel.: +46-(0)8-5177 0464 (R.Z.)

Supplementary table 1: Summary of BL subgroups [5-7]

**Endemic**	**Sporadic**	**Immunodeficiency-associated**
African children, 4-7 years<br>Associated with EBV<br>Involves: bones of the jaw and other facial bones, kidneys, gastrointestinal tract, ovaries, breast and other extranodal sites<br>The break point in *MYC* is < 100 kb upstream from exon 1, and the break point in the *IgH* is in the joining segment.	Worldwide, children and adults<br>Associated with EBV in 15-30% cases.<br>Involves: abdomen (ileocecal area), ovaries, kidneys, omentum, Waldeyer's ring, breasts, lymph nodes, pleural effusions or ascites<br>The break point in *MYC* is located between exon 1 and 2, and the break point in *IgH* is in the switch region.	Worldwide, young adults.<br>Very often present with HIV, also occurs in immuno-suppressed transplant recipients.<br>Involves: lymph nodes, bone marrow, extranodal sites most often in abdomen.<br>The break point in *MYC* is between exon 1 and 2, and the break point in *IgH* is in the switch region.

Supplementary table 2: plasmids pMycNHE+ and pMycNHE- and non-B- DNA and target sequence, respectively[2].

Plasmid	Sequence 5′-3′
pMycNHE+	tctcctccccaccttccccaccctccccaccctccccataagcgcccctcccgggttcccaaa**gcagagggcgtgggggaaaagaaaaaaga**tcct
pMycNHE-	gtgtacggtgggaggtctatataagcagagctggtttagtgaaccgtcagatccgctagcaaa**gcagagggcgtgggggaaaagaaaaaaga**tcca

[2] Non-B-DNA forming sequence is underlined and ON target sequence is marked with bold. pMycNHE- has a pEGFPLuc backbone. The target sequence is cloned downstream of CMV promoter. pMycNHE+ has a pBV-Luc backbone with inserted Myc promoter (pDel-1) [40]. The $NHEIII_1$ and the target sequence are cloned upstream of minP promoter.

CAGCAAATTGGGGGACTCAGTCTGGGTGGAAGGT|ATCCAATCCAGATAGCTGTGCATACATAATGCATAATA
CA

P0 ↱
TGACTCCCCCCAACAAA**T**GCAATGGGAGTTTATTCATAA|CGCGCTCTCCAAGTATACGTGGCAATGCGTTGC
TG

GGTTATTTTAATCATTCTAGGCATCGTTTTCCTCCTTATGCCTCTATCATTCCTCCCTATCTACACTAACATCCC

ACGCTCTGAACGCGCGCCCATTAATACCCTTCTTTCCTCCACT|CTCCCTGGGACTCTTGATCAAAGCGCGGC
CC

TTTCCCCAGCCTTAGCGAGGCGCCCTGCAGCCT|GGTACGCGCGTGG|CGTGGCGGTGGGCGCGCAGTGCG
TTCT

CGGTGTGGAGGGCAG|CTGTTCCGCCTGCGATGATTTATACTCACAGGACAAGGATGCGGTTTGTC|AAAC|A
GT

A|CTGCTA|CGGAGGAG|CAGCAGAGAAAGGG|A|GAGGGTTTGAG|AGGGAGCAAAAGAAAATGGTAGGCGCG
C

GTAGTTAATTCATGCGGCTC|TCTTACTCTGTTTACATCCTAGAGC|TAGAGTGCTC|GGCTGC|CCGGCTGAGT

C|TCCTCCCCACCTTCCCCACCCTCCCCACCCTCCCCATAAGCGCCCCTCCCGGGTTCCCAAAGCAGAGGG
CGTG

GGGGAAAAGAAAAAAGATCCTCTCT|CGCTAATCTCCGCCCACCGGCCCTTTATA|ATGCGAGGGT|CTGGAC
GG

P1 ↱
CTGAGG**A**CCCCCG|AGC|TGTGCT|GCTCGCGGCCGCCACCGCCGGGCCCCGGCC|GTCCCTGGCTCCCCT|
CCT

GCCTCGAGAAGGGCAGGGCTTCTCAGAGGCTTGGCGGGAAAAAGAACGGAGGGAGGGATCGCGCTGAGTA
TAAAA

P2 ↱
GCCGGTTTTCGGGGCTTTATCTA**A**CTCG

NNN = Tandem H-DNA or G-quadruplex forming sequence
| = Translocation point
↱ = promoter initiating site, base indicated in bold

Supplementary Figure 1. 127735336 to 127736236 Homo sapiens chromosome 8. [17,59]
